# Chromosomal abnormalities associated with mental retardation in female subjects

**DOI:** 10.4103/0971-6866.50867

**Published:** 2009

**Authors:** Samikshan Dutta, Jyothi Shaw, Swagata Sinha, Kanchan Mukhopadhyay

**Affiliations:** Manovikas Biomedical Research and Diagnostic Centre, 482, Madudah, Plot I-24, Sec.-J, E.M. Bypass, Kolkata - 700 107, India

**Keywords:** Chromosomal abnormalities, GTG-banding, karyotype, MR

## Abstract

Chromosomal abnormalities are thought to be the most common cause of mental retardation (MR). However, apart from a few selected types with typical aneuploidy, like Downs syndrome, Klinefelter syndrome, Turner syndrome, etc., the frequency of detectable chromosomal abnormalities in association with idiopathic MR is very low. In this study, we have investigated chromosomal abnormalities in female MR subjects (*n* = 150) by high-resolution GTG banding. Of them, 30 cases were diagnosed as Downs syndrome. Among the remaining (*n* = 120), chromosomal abnormalities/marked polymorphisms were detectable in only three MR cases (0.025).

## Introduction

Chromosomal abnormalities that alter developmental gene expression are the most common cause of mental retardation (MR).[1] Overall, ∼10% of MR cases suffer from chromosomal abnormalities.[2] In depth analysis has shown that nearly 40% severe MR cases suffer from some form of chromosomal abnormalities whereas only about 10% mild MR cases show detectable chromosomal aberrations.[3] Telomeric regions of chromosomes are the most gene-rich regions and any deletion or alteration in this region had been reported to account for nearly 2.5% MR, with or without dysmorphic features.[4] This percentage varies greatly between 2 and 29% (overall 6%). These regions are also highly susceptible to meiotic recombination and may be responsible for idiopathic MR (IMR).[5] Studies have also shown a male preponderance for all types of MR, with males being 1.6-1.7-times more vulnerable as compared with females,[6,7] which may be attributed to X-linked disorders. However, till date, not many studies have been carried out on the female MR probands. The present investigation was aimed at studying chromosomal abnormalities in female IMR subjects.

## Materials and Methods

Heparinized blood sample was collected for lymphocyte culture from 150 female MR cases after obtaining informed written consent for participation.[8] Plasma was added to an RPMI 1640 medium supplemented with 10% fetal bovine serum and phytohemagglutinin and was incubated for 69 h at 37°C in a CO_2_ incubator (Heraeus, Kendro Laboratory Products GmbH, Germany) followed by metaphase arrest with colcemid (N-deacetyl-N-methylcolchicine).

Cells were harvested after 45 min of colcemid treatment and subjected to hypotonic shock with 0.075 M KCl followed by fixation of the cell pellet with chilled Carnoy's fixative (methanol:aetic acid, 3:1). Later, GTG-banding analysis[9] of the fixed cells on glass slides was carried out with controlled trypsin digestion and Geimsa staining. Cells were visualized under the oil immersion lens of a Zeiss Axioskop2 plus microscope, Carl Zeiss India Pvt. Ltd. and at least 50 well-spread metaphase plates were analyzed with the karyoimager software.

DNA was isolated from ethylenediaminetetraacetic acid-treated blood samples and polymerase chain reaction (PCR) was carried out with SRY-specific primers for detection of Y chromosome (details available on request).

## Results

Of the 150 female MR probands recruited, highest frequency (0.2) of chromosomal abnormalities was observed for Downs syndrome (with trisomy 21), with only few cases of other abnormalities being reported [Table 1]. Of the remaining 120 cases, 64 were identified as IMR, with a very low frequency of detectable chromosomal abnormalities (0.046), and 56 cases were diagnosed as MR, with records of birth asphyxia, epileptic seizure, cerebral palsy, William syndrome (del 7q), Turner syndrome (XO), etc. The IMR cases showing abnormal karyotypes are discussed below.

**Table 1:** Chromosomal abnormalities detected during screening

Abnormalities	Type	No. of cases
Numerical variation	Trisomy 21, Turner syndrome	30, 1
Structural anomaly	Xq del, William syndrome (7q del)	1, 1
Polymorphic variation	22p, 15p	1, 1
Sex chromosomal abnormalities	46 XY female	1

## Case Reports

### Case 1

A 13-year-old girl presented with short stature, talkativeness, compulsion of washing hands repetitively, uncontrolled anger, scholastic backwardness, and amenorrhea. Detailed medical history of the patient showed that during the 7th month of gestation, the mother had an accident and the baby stopped movement. The child was prematurely (at the 7th month) delivered by induction (BW 2.5 kg). Her developmental milestones were normal. She had to undergo a surgery for congenital pyloric stenosis at the age of 1.5 months, had typhoid with measles at the 6th year, and duodenal ulcer at the 10th year. No familial history of amenorrhea was reported in the maternal side. Abdominal ultrasonogram revealed absence of clear ovaries and uterus. Mental health professionals made a diagnosis of obsessive-compulsive disorder with mild MR.

**Figure 1:**
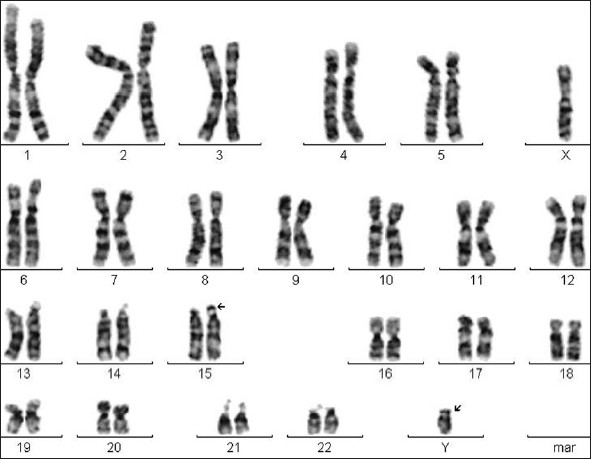
Karyotype of a 13-year-old girl with 46XY; 15ps+

**Figure 2:**
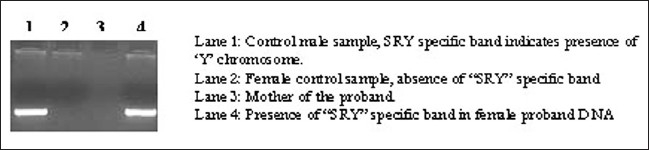
Detection of a Y chromosome-specific marker in the genomic DNA of case 1

High-resolution banding analysis revealed an apparent male karyotype of 46, XY with a polymorphic variation of enlarged satellites in the 15 ‘p’ arm (15ps+): the satellites on one homologue of chromosome 15 were enlarged [Figure 1]. This polymorphism was also detectable in the mother. Presence of the Y chromosome, or at least a part of it, was confirmed by PCR using SRY gene-specific primers [Figure 2]. Her testosterone level was 1.3 nmol/l. Because her testosterone level was within normal limits, whether this “Y” chromosome contributed to any major phenotype has not yet been resolved.

### Case 2

A 10-year-old girl with microcephaly, short stature, and speech articulation disorder was presented. Birth history showed that pregnancy was uneventful and an underweight child with cleft palate was delivered normally at the 10th month (BW 1.25 kg). The child suffered from birth asphyxia (∼8 min). She had a problem in breast feeding due to weakness and was under glucose supplementation for 15 days. She had measles (six times) during early childhood and global delay in developmental milestones. She was diagnosed as mild MR. Her mother was apparently normal but was found to have microcephaly, like her daughter.

**Figure 3:**
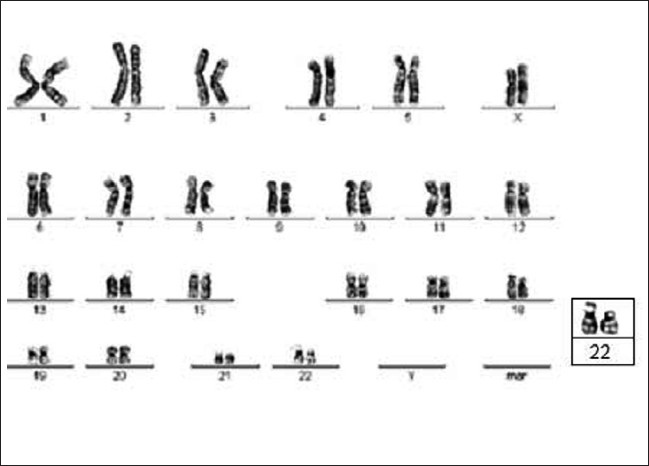
Karyotype of case 2 showing 46XX; 22ps+

In this case, the satellite on one homologue of chromosome 22 was prominent (22ps+), which was also fFound to be present in her mother [Figure 3]. Although her mother had a normal IQ, the proband had a low IQ (< 70) and delayed brain development.

### Case 3

An 18-year-old girl was referred for scholastic backwardness. She was tall, thin, with elongated facial features and a hoarse voice. Physical examination revealed clitoromegaly and hirsutism. Her birth history was uneventful and delivery was normal. Developmental history was at par with age. Psychological assessment revealed that the girl had mild MR with normal thyroid status. She was hospitalized at the 16th year for abdominal pain and amenorrhea. Abdominal ultrasonogram revealed a pelvic mass extending above the umbilicus and left ovarian tumor. Her right ovarian surface looked partly bosselated. Ovariectomy was performed. Histopathology did not reveal presence of testicular tissue.

**Figure 4:**
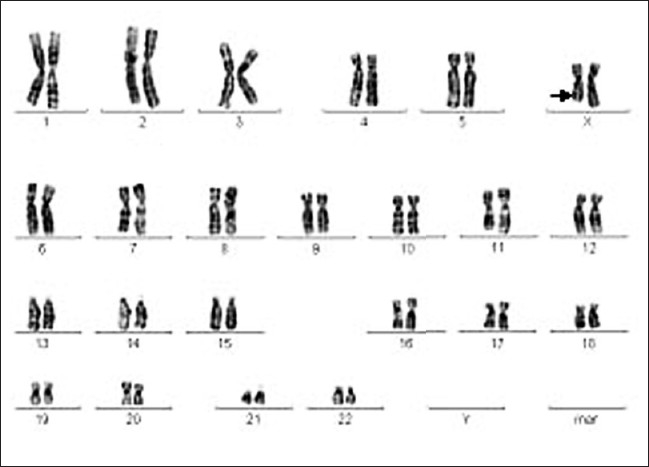
Karyotype of case 3; 46XX; del Xq27

In the proband, a deletion in one of the X chromosomes, Xq27, was observed [Figure 4]. The deletion of the terminal X region could be responsible for altered gene expression leading to virilization in the patient. On the other hand, this might be due to inactivation of Xq in the intact X chromosome (one without deletion).

## Discussion

As was suggested previously, interpretation of subtelomeric rearrangements or other chromosomal abnormalities in MR individuals is often complicated by the fact that many deletions or duplications appear to be benign in familial variants while associated with a particular phenotype in an affected individual.[5] The variation in the expression pattern observed in the present investigation between the probands and their mothers also supports the above notion. Epigenetic regulation[10] or environmental factors may give rise to such differences in phenotypic expression.

Other than these three, no other chromosomal abnormality was noted in the cases studied. It may be assumed that minute telomeric or subtelomeric changes were overlooked by GTG-banding analysis. More detailed analysis employing special techniques like subtelomere fluorescent *in situ* hybridization, comparative genomic hybridization, or spectral karyotyping may help in determining the actual frequency of chromosomal abnormalities in the MR cases.
